# Proliferator-activated receptor gamma Pro12Ala interacts with the insulin receptor substrate 1 Gly972Arg and increase the risk of insulin resistance and diabetes in the mixed ancestry population from South Africa

**DOI:** 10.1186/1471-2156-15-10

**Published:** 2014-01-21

**Authors:** Zelda Vergotine, Yandiswa Y Yako, Andre P Kengne, Rajiv T Erasmus, Tandi E Matsha

**Affiliations:** 1 Biomedical Sciences, Faculty of Health and Wellness Sciences, Cape Peninsula University of Technology, PO Box 1906, Bellville 7530, Cape Town, South Africa; 2 Division of Chemical Pathology, Stellenbosch University, Cape Town, South Africa; 3 NCRP for Cardiovascular and Metabolic Diseases, South African Medical Research Council, Cape Town, South Africa; 4 Department of Medicine, University of Cape Town, Cape Town, South Africa

**Keywords:** *IRS1* Gly972Arg, *PPARG* Pro12Ala, Insulin resistance, Type 2 diabetes, Africa

## Abstract

**Background:**

The peroxisome proliferator-activated receptor gamma (*PPARG*), Pro12Ala and the insulin receptor substrate (*IRS1*), Gly972Arg confer opposite effects on insulin resistance and type 2 diabetes mellitus (T2DM). We investigated the independent and joint effects of *PPARG* Pro12Ala and *IRS1* Gly972Arg on markers of insulin resistance and T2DM in an African population with elevated risk of T2DM. In all 787 (176 men) mixed-ancestry adults from the Bellville-South community in Cape Town were genotyped for *PPARG* Pro12Ala and *IRS1* Gly972Arg by two independent laboratories. Glucose tolerance status and insulin resistance/sensitivity were assessed.

**Results:**

Genotype frequencies were 10.4% (*PPARG* Pro12Ala) and 7.7% (*IRS1* Gly972Arg). Alone, none of the polymorphisms predicted prevalent T2DM, but in regression models containing both alleles and their interaction term, *PPARG* Pro12 conferred a 64% higher risk of T2DM. Furthermore *PPARG* Pro12 was positively associated in adjusted linear regressions with increased 2-hour post-load insulin in non-diabetic but not in diabetic participants.

**Conclusion:**

The *PPARG* Pro12 is associated with insulin resistance and this polymorphism interacts with *IRS1* Gly972Arg, to increase the risk of T2DM in the mixed-ancestry population of South Africa. Our findings require replication in a larger study before any generalisation and possible application for risk stratification.

## Background

Insulin resistance is a fundamental etiopathogenic factor for type 2 diabetes and is also linked to a wide array of other pathophysiological derangements including hypertension, hyperlipidemia, atherosclerosis and polycystic ovarian disease [1]. The gold standard method for assessing insulin resistance/sensitivity is the euglycemic hyperinsulinemic clamp [2,3], however, this technique is cumbersome, particularly for large scale epidemiological studies. Thus relatively simple, non-invasive alternative techniques validated against the euglycemic clamp have been proposed. The homeostatic model assessment of insulin resistance (HOMA-IR) [4] and quantitative insulin*-*sensitivity check index (QUICKI) [5] methods are commonly used for insulin resistance and insulin sensitivity, respectively. It is well recognised that the development of insulin resistance and type 2 diabetes is in part modulated by the gene-gene interaction processes.

The peroxisome proliferator-activated receptor gamma (*PPARG*) and the insulin receptor substrate (*IRS1*) genes have been shown to be associated with both insulin resistance and type 2 diabetes [6-11]. The *PPARG* is a member of the super family of nuclear receptors reported to be involved in the regulation of adipocyte differentiation [12], lipid metabolism and insulin sensitivity [6]. Several variants in the *PPARG* gene have been identified, with the most prevalent variant being the Pro12Ala polymorphism resulting from the CCA-to-GCA missense mutation in codon 12 of exon B that encodes the NH_2_ terminal residue [13-15]. The proline which is the common allele is associated with increased risk whilst the alanine confers a protective effect against insulin resistance and type 2 diabetes [9,16-20]. In contrast, the glycine to arginine substitution in codon 972 (Gly972Arg) of the *IRS1* gene is associated with an increased risk of insulin resistance [21]. In view of the above, we investigated the independent and joint effects of *PPARG* Pro12Ala and *IRS1* Gly972Arg on markers of insulin resistance and type 2 diabetes in the mixed-ancestry population of South Africa, a population with elevated risk of type 2 diabetes.

## Results

Clinical characteristics of participants overall and according to type 2 diabetes status are summarized in Table 1 indicating that two hundred and twelve participants (26.9%) had type 2 diabetes. As expected, the distribution of the level of insulin resistance/sensitivity indicators was significantly different between the two groups (all p < 0.0001, except for glucose/insulin ratio (p = 0.016). Furthermore, compared with non-diabetic participants, those with type 2 diabetes had significantly higher levels of adipometric variables (all p ≤ 0.028), systolic blood pressure (p < 0.0001), triglycerides (p < 0.0001), GGT and CRP (both p < 0.0001), whilst eGFR (p = 0.015) and HDL cholesterol (p = 0.0001) were significantly lower.

**Table 1:** General characteristics of the overall population and by diabetic status

**Variable**	**Non-diabetic**	**Diabetic**	**P-value**	**Overall**
Number	575	212		787
Gender, male n (%)	131 (22.8)	45 (21.2)	0.642	176
Mean age, year (SD)	51.3 (15.5)	59.3 (13.4)	<0.0001	53.5 (15.4)
Mean systolic blood pressure, mmHg (SD)	123 (19)	131 (23)	<0.0001	124 (21)
Mean diastolic blood pressure, mmHg (SD)	75 (12)	78 (15)	0.035	76 (13)
Hypertension, n (%)	306 (53.2)	138 (65.1)	0.003	444
Mean body mass index, kg/m^2^ (SD)	29.1 (7.1)	31.7 (7.2)	<0.0001	29.8 (7.2)
Mean waist circumference, cm (SD)	95 (15)	102 (14)	<0.0001	97 (16)
Mean hip circumference, cm (SD)	109 (14)	111 (15)	0.028	109 (14)
Mean waist/hip ratio, (SD)	0.87 (0.10)	0.92 (0.09)	<0.0001	0.88 (0.10)
Mean HbA1c, % (SD)	5.7 (0.4)	7.8 (2.1)	<0.0001	6.3 (1.5)
Mean HbA1c, mmol/mol (SD)	39 (4.4)	62 (23)	<0.0001	45 (16.4)
Mean fasting blood glucose, mmol/l (SD)	5.1 (0.7)	9.8 (4.4)	<0.0001	6.4 (3.1)
Mean 2 h glucose, mmol/l (SD)	6.4 (1.6)	13.4 (5.3)	<0.0001	7.3 (3.5)
Mean eGFR, ml/min (SD)	76.0 (21.1)	71.2 (25.2)	0.015	74.7 (22.4)
Mean triglycerides, mmol/l (SD)	1.4 (0.9)	1.7 (0.9)	<0.0001	1.5 (0.9)
Mean HDL cholesterol, mmol/l (SD)	1.3 (0.4)	1.2 (0.3)	0.0001	1.3 (0.4)
Mean LDL cholesterol, mmol/l (SD)	3.6 (1.0)	3.7 (1.1)	0.191	3.6 (1.0)
Mean total cholesterol, mmol/l (SD)	5.5 (1.2)	5.7 (1.3)	0.070	5.6 (1.2)
Median GGT (25th-75th percentiles)	26 [18-39]	31 [23-39]	<0.0001	27 [19-42]
Median CRP (25th-75th percentiles)	3.4 [0.8-8.4]	5.2 [1.9-10.8]	<0.0001	4.0 [1.1-9.4]
Median insulin mmol/l (25th-75th percentiles)	6.9 [3.3-12.5]	9.2 [3.7-16.6]	0.0009	7.5 [3.3-13.5]
Median 2 h insulin mmol/l (25th-75th percentiles)	35.3 [19.2-64.5]	58.9 [22.1-115.2]	0.0009	36.8 [19.5-72.7]
Median glucose/insulin (25th-75th percentiles)	0.72 [0.42-1.51]	0.88 [0.50-2.30]	0.016	0.75 [0.43-1.68]
Median HOMA-IR (25th-75th percentiles)	1.6 [0.7-2.9]	3.5 [1.5-6.7]	<0.0001	1.9 [0.8-3.7]
Median HOMA-B% (25th-75th percentiles)	90.0 [41.1-160.0]	40.7 [12.4-77.8]	<0.0001	71.2 [28.6-44.9]
Median QUICKI (25th-75th percentiles)	0.15 [0.14-0.18]	0.14 [0.13-0.15]	<0.0001	0.15 [0.14-0.17]
Median FIRI (25th-75th percentiles)	1.4 [0.6-2.6]	3.1 [1.3-6.0]	<0.0001	1.8 [0.7-3.3]
Median 1/HOMA-IR (25th-75th percentiles)	0.64 [0.34-1.49]	0.29 [0.15-0.66]	<0.0001	0.54 [0.27-1.26]

CRP, C-reactive protein; eGFR, estimated glomerular filtration rate; FIRI, fasting *insulin* resistance index; GGT, γ-glutamyltransferase; HbA1c, glycated haemoglobin; HDL, High Density Lipoproteins; HOMA-β%, functional β-cells; HOMA-IR, homeostatic model assessment of insulin resistance; LDL, Low Density Lipoproteins; QUICKI, the quantitative *insulin*-sensitivity check index; SD, standard deviation.

*IRS1* Gly972Arg and *PPARG* Pro12Ala variants were in HWE (p > 0.05) and their genotype and allele distribution by type 2 diabetes status is summarized in Table 2. Overall, the genotype distributions of the two polymorphisms did not differ significantly between the two groups. However the allele G of *PPARG* (12Ala) was significantly more frequent in the diabetic subjects than in the non-diabetic subjects (13.7% vs. 9.3%, p = 0.012). The genotype frequencies, *PPARG* Pro12Ala and *IRS1* Gly972Arg were 10.4% and 7.7%, respectively.

**Table 2:** Genotype distributions, minor allele frequencies and unadjusted p-values for comparing genotype distribution according to diabetes status, additive allelic effects between diabetes groups

	**Non-diabetic**	**Diabetic**	**p-value**	**Overall**
N	575	212		787
***IRS1***				
G/G, n (%)	526 (91.5)	199 (93.9)	0.485	725 (92.1)
G/A, n (%)	48 (8.3)	13 (6.1)		61 (7.7)
A/A, n (%)	1 (0.2)	0 (0)		1 (0.1))
A, n (%)	50 (4.4)	13 (3.1)	0.131	63 (4.0)
HWE (p-value)	>0.999	>0.999		>0.999
***PPARG***				
C/C, n (%)	521 (90.6)	183 (86.3)	0.161	704 (89.4)
C/G, n (%)	53 (9.2)	29 (13.7)		82 (10.4)
G/G, n (%)	1 (0.2)	0 (0)		1 (0.1)
G, n (%)	55 (4.9)	29 (6.8)	0.012	84 (5.3)
HWE (p-value)	>0.999	0.605		0.719

HWE, Hardy-Weinberg Equilibrium (HWE p-values are from exact tests). IRS1, Insulin Substrate Receptor 1; PPARG, Peroxisome Proliferator-Activated Receptor Gamma.

In generalised linear regression analyses adjusted for age, sex and type 2 diabetes (Table 3), the *IRS1* allele A (972Arg) was associated with none of the marker of glycaemia, insulin resistance or insulin sensitivity, both overall and in participants with and without type 2 diabetes taken separately; with no evidence of significant statistical interaction by type 2 diabetes status (all interaction p ≥ 0.330), except for 2 hour glucose where the effect size appeared to be greater although non-significantly among diabetic than non-diabetic participants (interaction p = 0.038). In similar generalised linear regression models (Table 3) the *PPARG* allele C (Pro12) increased 2 hour insulin levels in the overall cohort (p = 0.009) and in the non-diabetic group only (p = 0.0003) after stratification by type 2 diabetes status, with a significant statistical interaction (p = 0.017). Otherwise, the *PPARG* allele C was not significantly associated with the marker of glycaemia, insulin resistance or insulin sensitivity, both overall and by type 2 diabetes status; with evidence however that the effect on 2-hour glucose if any, could be more pronounced in people with type 2 diabetes (p-value = 0.002 for the *PPARG* allele C type 2 diabetes interaction). The main effects for *IRS1* and *PPARG* did not change significantly when they were adjusted for each other in regression models with or without further adjustment for their interaction term.

**Table 3:** Generalized linear regression models showing the effects of genes on markers of insulin resistance/sensitivity

		**Non- diabetic**			**Diabetic**			**p interaction**
**Allele**	**Phenotype**	**Effects size**	**95% CI**	**p**	**Effects size**	**95% CI**	**p**	
***IRS1 *** **A**	Fasting glucose	0.09	−0.11 to 0.30	0.372	−0.53	−2.96 to 1.90	0.668	0.330
	2 h glucose	0.08	−0.36 to 0.51	0.723	2.33	−2.09 to 6.75	0.304	0.038
	HbA1c	0.02	−0.10 to 0.14	0.746	0.13	−1.04 to 1.31	0.823	0.853
	Fasting insulin	−1.02	−3.76 to 1.71	0.463	−3.27	−20.75 to 14.21	0.714	0.690
	2 h insulin	1.62	−15.50 to 18.75	0.853	2.36	−63.13 to 67.86	0.944	0.944
	Glucose/insulin	−0.14	−2.26 to 1.97	0.894	0.30	−8.40 to 9.00	0.946	0.896
	HOMA-IR	−0.22	−0.87 to 0.42	0.498	−2.03	−9.96 to 5.91	0.617	0.448
	QUICKI	0.002	−0.015 to 0.018	0.857	−0.001	−0.025 to 0.022	0.903	0.877
	FIRI	−0.20	−0.78 to 0.38	0.480	−1.82	−8.97 to 5.31	0.617	0.448
***PPARG *** **C**	Fasting glucose	−0.06	−0.26 to 0.13	0.528	0.003	−1.71 to 1.72	0.995	0.970
	2 h glucose	−0.02	−0.44 to 0.41	0.933	−2.46	−5.68 to 0.76	0.137	0.002
	HbA1c	0.07	−0.04 to 0.19	0.216	−0.18	1.02 to 0.65	0.663	0.362
	Fasting insulin	−0.56	−3.21 to 2.09	0.617	−5.72	−18.00 to 6.57	0.363	0.283
	2 h insulin	34.0	15.9 to 52.2	0.0003	−14.4	−64.7 to 35.8	0.575	0.017
	Glucose/insulin	−0.21	−2.26 to 1.83	0.840	−2.62	−8.82 to 3.37	0.407	0.307
	HOMA-IR	−0.12	0.74 to 0.51	0.714	−2.31	−7.89 to 3.27	0.417	0.299
	QUICKI	0.0001	−0.015 to 0.016	0.924	−0.007	−0.024 to 0.009	0.377	0.481
	FIRI	−0.10	−0.67 to 0.46	0.714	−2.08	−7.10 to 2.94	0.417	0.299

Models are adjusted for age, sex and diabetes. FIRI, fasting insulin resistance index; HOMA-IR, homeostatic model assessment of insulin resistance; IRS1, Insulin Substrate Receptor 1; PPARG, Peroxisome Proliferator-Activated Receptor Gamma; QUICKI, the quantitative insulin-sensitivity check index.

In logistic regression models adjusted for each other, or containing age and sex, with and without further adjustment for markers of insulin resistance/sensitivity (Table 4), neither the *IRS1* allele A, nor the *PPARG* was significantly associated with prevalent type 2 diabetes. However, in the model containing both alleles and their interaction term, the *PPARG* allele C was associated with higher risk of prevalent type 2 diabetes, odds ratio (95% confidence interval) 1.64 (1.00-2.64).

**Table 4:** **Odds ratio and 95**% **confidence intervals from logistic regression for the prediction of diabetes**

**Allele**	**Covariates**	**OR (95% CI)**	**P**
***IRS1 *** **A**	Gene alone	0.69 (0.36-1.25)	0.250
	Sex, age	0.67 (0.34-1.24)	0.228
	Sex, age, insulin	0.70 (0.35-1.31)	0.290
	Sex, age, 2 h insulin	0.76 (0.28-1.76)	0.562
	Sex, age, HOMA-IR	0.72 (0.35-1.39)	0.347
	Sex, age, QUICKI	0.70 (0.35-1.31)	0.285
	Sex, age, FIRI	0.72 (0.35-1.39)	0.347
	Sex, age, glucose/insulin	0.67 (0.34-1.27)	0.224
	*PPARG*	0.69 (0.36-1.25)	0.248
	*PPARG*, IRS1*PPARG	0.85 (0.43-1.57)	0.613
***PPARG *** **C**	Gene alone	1.48 (0.91-2.36)	0.104
	Sex, age	1.40 (0.85-2.28)	0.176
	Sex, age, fasting insulin	1.49 (0.90-2.45)	0.116
	Sex, age, 2 h insulin	1.24 (0.56-2.53)	0.571
	Sex, age, HOMA-IR	1.51 (0.88-2.56)	0.131
	Sex, age, QUICKI	1.40 (0.83-2.32)	0.198
	Sex, age, FIRI	1.51 (0.88-2.56)	0.131
	Sex, age, glucose/insulin	1.41 (0.85-2.51)	0.174
	*IRS1*	1.48 (0.91-2.37)	0.103
	*IRS1*, IRS1*PPARG	1.64 (1.00-2.64)	0.046

*, interaction; FIRI, fasting insulin resistance index; HOMA-IR, homeostatic model assessment of insulin resistance; IRS1, Insulin Substrate Receptor 1; PPARG, Peroxisome Proliferator-Activated Receptor Gamma; QUICKI, the quantitative *insulin*-sensitivity check index.

## Discussion

The mixed ancestry population of South Africa has one of the highest prevalence of type 2 diabetes in South Africa and sub-Saharan Africa at large [22], however, genetic abnormalities that can fully account for this have not been identified. In this study, we show that *PPARG* Pro12 is significantly associated with insulin resistance and type 2 diabetes in this population. We observed that neither *IRS1* 972Arg allele nor *PPARG* 12Ala were associated with type 2 diabetes or insulin resistance/sensitivity, but in a model containing both the alleles and their interaction term, the presence of the *PPARG* Pro12 conferred a 64% risk of prevalent type 2 diabetes. Furthermore the *PPARG* Pro12 was associated with increased levels of 2 hour post-OGTT insulin. Overall, our findings convincingly demonstrate that *PPARG* Pro12Ala –*IRS1* Gly972Arg interactions, *PPARG* Pro12 and susceptibility to environmental factors might modulate the relationship between insulin resistance and type 2 diabetes in this population.

The gene-gene interaction between *IRS1* Gly972Arg and *PPARG* Pro12Ala is of interest because the two polymorphisms exert opposite effects on type 2 diabetes predispositions. The Gly972Arg is a functional polymorphism reported to impair insulin signaling in transfected cell lines and in human cells carrying the variant [23-25]. Although individuals carrying the Gly972Arg are reported to have a 25% increased risk for developing diabetes [10], genome wide association (GWAS) studies involving subjects of European descent found no association between *IRS1* and type 2 diabetes [26,27]. On the other hand, the *PPARG* Pro12Ala, particularly the 12Ala has been associated with a reduced risk of type 2 diabetes and insulin resistance [9,16-20]. As such, the polymorphisms of the *IRS1* and *PPARG* genes have been shown to interact and elevate insulin sensitivity. This was evident in a study done by Stumvoll *et al*., [28] where the authors showed that insulin sensitivity was significantly greater in subjects with X/Ala (PPARγ2) + X/Arg (*IRS1* 972) than in subjects with Pro/Pro (PPARγ2) + X/Arg (*IRS1*) while no differences were observed in X/Ala (PPARγ2) + Gly/Gly (*IRS1* 972) and Pro/Pro (PPARγ2) + Gly/Gly (*IRS1* 972) carriers [28]. Similarly, the interaction between the two polymorphisms has been associated with higher adiponectin levels and the greatest increase was found in subjects who were homozygous for both *PPARG* alanine (Ala12Ala) and *IRS1* glycine (Gly972Gly) [29]. Adiponectin is secreted by the adipose tissue and is inversely associated with obesity, insulin resistance, type 2 diabetes and cardiovascular disease [30,31]. Taken together these reports including ours confirm the combined effect of the two SNPs on insulin resistance and type 2 diabetes.

Several epidemiological studies have demonstrated that *PPARG* Pro12Ala is associated with insulin sensitivity and diabetes mellitus [6-9]. In the Human Genome Epidemiology (HuGE) meta-analysis involving 32 849 type 2 diabetes cases and 47 456 controls, the Pro12Ala was associated with a 14% lower risk for developing type 2 diabetes [8]. However, other investigators have failed to demonstrate an association between Pro12Ala and insulin sensitivity using the gold standard method for assessing insulin resistance/sensitivity, the euglycemic hyperinsulinemic clamp [32,33]. The differences between studies have been attributed to body mass index and ethnic differences [7,8]. The frequency of the 12Ala has been reported to be more frequent in Caucasians than in Asian populations [8], but conferred significantly greater protection against type 2 diabetes among Asians than Caucasians (35% vs. 15%) [7]. However, when the authors adjusted for body mass index the differences were no longer significant [7]. In our study, the 10.4% frequency of Pro12Ala polymorphism is comparable to that found in Caucasians and the Pro12 was strongly associated with an increased 2 hour post-OGTT insulin levels in non-diabetic subjects. Our results further add to the growing body of evidence on the association of *PPARG* Pro12Ala, insulin resistance and subsequent type 2 diabetes. Herein we investigated a heterogeneous population, with 32-43% Khoisan, 20 – 36% Bantu-speaking African, 21 – 28% European and 9 – 11% Asian ancestry [34]. Our present findings require replication in a larger study involving other homogenous population before they can be considered as established in Africa.

The strengths of the present study include the use of both fasting and OGTT derived indices for assessing type 2 diabetes and insulin resistance. OGTT derived indices have been found to be of superior predictive power to simple fasting indices of IR as they take post-load glucose-insulin interaction into account [35]. Furthermore, we made use of two independent laboratories to genotype our study population. The major limitation of our study is the statistical power of the study which was limited by the small sample size and the examination of gene-gene interaction effects reduced the sample further. In addition, we did not adjust for population stratification. Potential population stratification in unrelated sample may cause spurious positive or negative associations in population-based association studies [36]. To minimise this type of confounding in association studies, several approaches have been suggested that utilise specific informative markers and loci to model ancestral differences between cases and controls and subsequently correct allele frequency variations at candidate loci in populations. However, markers suitable for mapping disease genes or correcting for population stratification in the mixed ancestry are not yet available. Lastly, the nature of this study is cross-sectional with high female to male participation, the latter being a common trend in South African population studies.

## Conclusion

Despite the above mentioned limitations, our results provide the first preliminary evidence for genetic predisposition to insulin resistance and subsequent type 2 diabetes in an African population with a high prevalence of type 2 diabetes. In conclusion, the *PPARG* Pro12 is associated with insulin resistance and this polymorphism interacts with an additional unfavourable genetic polymorphism, *IRS1* Gly972Arg, to increase the risk of type 2 diabetes in the mixed ancestry population of South Africa.

## Methods

### Study setting and population

The study setting, survey design and procedures have been described in details elsewhere [22,37]. Briefly, participants were members of a cohort study conducted in Bellville-South, Cape Town. According to the 2011 South African population census, the population is predominantly of mixed ancestry (76%) followed by black Africans (18.5%) and Caucasian and Asians making only 1.5%. Eligible participants were invited to take part in a community based survey from January 2008 to March 2009 (Cohort 1), and January 2011 to November 2011 (Cohort 2). The study was approved by the Research Ethics Committees of the University of Stellenbosch (HREC Ref No: N09/05/146) and Cape Peninsula University of Technology, Faculty of Health and Wellness Sciences ethics committee (Reference Number: CPUT/HW-REC 2008/002 and CPUT/HW-REC 2010). The study was conducted according to the Code of Ethics of the World Medical Association (Declaration of Helsinki). All participants signed written informed consent after all the procedures were fully explained in the language of their choice.

### Clinical data

All consenting participants received a standardized interview and physical examination during which blood pressure was measured according to the World Health Organisation (WHO) guidelines [38] using a semi-automated digital blood pressure monitor (Rossmax PA, USA) on the right arm in a sitting position. Other clinical measurements included the body weight, height, waist and hip circumferences. Weight (to the nearest 0.1 kg) was determined in a subject wearing light clothing and without shoes and socks, using a Sunbeam EB710 digital bathroom scale, which was calibrated and standardized using a weight of known mass. Waist circumference was measured using a non-elastic tape at the level of the narrowest part of the torso, as seen from the anterior view. The hip circumference was also measured using a non-elastic tape around the widest portion of the buttocks. All anthropometric measurements were performed three times and their average used for analysis. Participants with no history of doctor diagnosed diabetes mellitus underwent a 75 g oral glucose tolerance test (OGTT) as recommended by the WHO [39].

### Laboratory measurements

Blood samples were collected after an overnight fast and processed for further biochemical analysis. Plasma glucose was measured by enzymatic hexokinase method (Cobas 6000, Roche Diagnostics, Germany) and glycated haemoglobin (HbA1c) by turbidimetric inhibition immunoassay (Cobas 6000, Roche Diagnostics, Germany) this being a National Glycohaemoglobin Standardisation Programme (NGSP) certified method. Creatinine levels were measured using the standardized creatinine assay (Cobas 6000, Roche Diagnostics, Germany). Total cholesterol (TC), high density lipoprotein cholesterol (HDL-c), triglycerides (TG) and γ-glutamyltransferase (GGT) were estimated by enzymatic colorimetric methods (Cobas 6000, Roche Diagnostics). Low density lipoprotein cholesterol (LDL-c) was calculated using Friedewald’s formula [40]. Insulin was determined by a microparticle enzyme immunoassay (Axsym, Abbot). C-reactive protein (CRP) was measured by a high-sensitivity CRP assay, based on the highly sensitive Near Infrared Particle Immunoassay rate methodology (Immage® Immunochemistry System; Beckman Coulter), with a lower limit of detection of 0.2 mg/L.

### SNP genotyping

Genomic DNA was extracted from whole blood samples collected in an EDTA tube. Single nucleotide polymorphisms (SNPs) in the *IRS1* (rs1801278, G > A) [GeneBank: NM_005544], and *PPARG* (rs1801282, C > G) [GeneBank: NM_015869] were genotyped using high throughput real-time polymerase chain reaction (RT-PCR) in two independent laboratories (Centre for Proteomic and Genomic Research Institute of infectious Diseases and Molecular Medicine, Faculty of Health Sciences, University of Cape Town and Obesity and Chronic Diseases of Lifestyle, Faculty of Health & Wellness Sciences, Cape Peninsula University of Technology) on the ABI Prism 7900HT platform(Applied Biosystems, USA) and a BioRad Optica (BioRad, USA) using Taqman genotyping assay (Applied Biosystems, USA). Direct sequencing was used to for analytical validation of high throughput genotyping against direct sequencing as the gold standard

### Definitions and calculations

Body mass index (BMI) was calculated as weight per square meter (kg/m2) and waist-hip-ratio (WHR) as waist/hip circumferences (cm). Type 2 diabetes status was based on a history of doctor-diagnosis, a fasting plasma glucose ≥7.0 mmol/l and/or a 2-hour post-OGTT plasma glucose ≥11.1 mmol/l. The homeostatic model assessment of insulin resistance (HOMA-IR) was calculated according to the formula: HOMA-IR = [fasting insulin concentration (mIU/L) × fasting plasma glucose (mmol/L]/22.5; while functional β-cells (HOMA-B%) were estimated using the formula: 20 × fasting insulin (μIU/ml)/fasting glucose (mmol/ml) − 3.5. The fasting insulin resistance index (FIRI) was calculated with the formula: [fasting insulin (μU/ml) × fasting glucose (mM)]/25 and the quantitative insulin-sensitivity check index (QUICKI) as: 1/[log (fasting insulin (μU/ml)) × log (fasting glucose (mg/dl)]. Glomerular filtration rate (GFR) was estimated by the 4-variable Modification of Diet in Renal Disease (MDRD) equation [41,42] applicable to standardised serum creatinine values.

### Statistical analysis

Of the 946 participants who took part in the survey, 941 consented for genetic studies. Among the latter, 154 were excluded for missing data on the genetic variables. Therefore, 787 had valid data for the current analyses. General characteristics of the study group are summarized as count and percentage for dichotomous traits, mean and standard deviation (SD) or median and 25th-75th percentiles for quantitative traits. Traits were log-transformed to approximate normality, where necessary, prior to analysis. SNPs were tested for departure from Hardy-Weinberg Equilibrium (HWE) expectation via a chi square goodness of fit test. Linear regression models were used for the analysis of quantitative traits and logistic regression models for dichotomous traits, always assuming additive models for the SNPs. Using linear and logistic models enabled us to adjust all analyses for known confounders as specified everywhere in the results. We investigated the additive allelic association of each SNP with each trait, overall and according to type 2 diabetes status, and tested for heterogeneity by adding the interaction term of type 2 diabetes and each SNP to a model that contained the main effects of type 2 diabetes and the relevant SNP. Results corresponding to p-values below 5% are described as significant. We did not adjust for multiple testing. All analyses used the statistical package R (version 3.0.0 [2013-04-03], The R Foundation for statistical computing, Vienna, Austria).

## Authors’ contribution

ZV: acquisition of data, preparation of the first draft and approval of final draft. YYY: acquisition of data and approval of final draft. APK: analysis and interpretation of data, revision for important intellectual content and approval of final draft. RTE: conception and design, revision for important intellectual content and approval of final draft. TEM: conception and design, acquisition and interpretation of data, preparation of the first draft and approval of final draft. All authors read and approved the final manuscript.

## References

[B1] Reaven GM Banting lecture 1988 role of insulin resistance in human disease Nutrition 1997 13 64 66 10.1016/s0899-9007(96)00380-2 9058458

[B2] Polonsky KS Given BD Hirsch L Shapiro ET Tillil H Beebe C Galloway JA Frank BH Karrison T Van-Cauter E Quantitative study of insulin secretion and clearance in normal and obese subjects J Clin Invest 1988 81 435 441 10.1172/JCI113338 3276729 PMC329588

[B3] DeFronzo RA Tobin JD Andres R Glucose clamp technique: a method for quantifying insulin secretion and resistance Am J Physiol 1979 237 E214 223 382871 10.1152/ajpendo.1979.237.3.E214

[B4] Matthews DR Hosker JP Rudenski AS Naylor BA Treacher DF Turner RC Homeostasis model assessment: insulin resistance and beta-cell function from fasting plasma glucose and insulin concentrations in man Diabetologia 1985 28 412 419 10.1007/BF00280883 3899825

[B5] Katz A Nambi SS Mather K Baron AD Follmann DA Sullivan G Quon MJ Quantitative insulin sensitivity check index: a simple, accurate method for assessing insulin sensitivity in humans J Clin Endocrinol Metab 2000 85 2402 2410 10902785 10.1210/jcem.85.7.6661

[B6] Buzzetti R Petrone A Ribaudo MC Alemanno I Zavarella S Mein CA Maiani F Tiberti C Baroni MG Vecci E Arca M Leonetti F Di Mario U The common PPAR-gamma2 Pro12Ala variant is associated with greater insulin sensitivity Eur J Hum Genet 2004 12 1050 1054 10.1038/sj.ejhg.5201283 15367918

[B7] Ludovico O Pellegrini F Di Paola R Minenna A Mastroianno S Cardellini M Marini MA Andreozzi F Vaccaro O Sesti G Trischitta V Heterogeneous effect of peroxisome proliferator-activated receptor gamma2 Ala12 variant on type 2 diabetes risk Obesity (Silver Spring) 2007 15 1076 1081 10.1038/oby.2007.617 17495182

[B8] Gouda HN Sagoo GS Harding AH Yates J Sandhu MS Higgins JP The association between the peroxisome proliferator-activated receptor-gamma2 (PPARG2) Pro12Ala gene variant and type 2 diabetes mellitus: a HuGE review and meta-analysis Am J Epidemiol 2010 171 645 655 10.1093/aje/kwp450 20179158 PMC2834889

[B9] Trombetta M Bonetti S Boselli ML Miccoli R Trabetti E Malerba G Pignatti PF Bonora E Del Prato S Bonadonna RC PPARG2 Pro12Ala and ADAMTS9 rs4607103 as "insulin resistance loci" and "insulin secretion loci" in Italian individuals. The GENFIEV study and the verona newly diagnosed type 2 diabetes study (VNDS) 4 Acta Diabetol 2013 50 401 408 10.1007/s00592-012-0443-9 23161442

[B10] Jellema A Zeegers MP Feskens EJ Dagnelie PC Mensink RP Gly972Arg variant in the insulin receptor substrate-1 gene and association with Type 2 diabetes: a meta-analysis of 27 studies Diabetologia 2003 46 990 995 10.1007/s00125-003-1126-4 12819898

[B11] Sesti G Federici M Hribal ML Lauro D Sbraccia P Lauro R Defects of the insulin receptor substrate (IRS) system in human metabolic disorders FASEB J 2001 15 2099 2111 10.1096/fj.01-0009rev 11641236

[B12] Evans RM Barish GD Wang YX PPARs and the complex journey to obesity Nat Med 2004 10 355 361 10.1038/nm1025 15057233

[B13] Auwerx J PPARgamma, the ultimate thrifty gene Diabetologia 1999 42 1033 1049 10.1007/s001250051268 10447513

[B14] Vigouroux C Fajas L Khallouf E Meier M Gyapay G Lascols O Auwerx J Weissenbach J Capeau J Magré J Human peroxisome proliferator-activated receptor-gamma2: genetic mapping, identification of a variant in the coding sequence, and exclusion as the gene responsible for lipoatrophic diabetes Diabetes 1998 47 490 492 10.2337/diabetes.47.3.490 9519760

[B15] Yen CJ Beamer BA Negri C Silver K Brown KA Yarnall DP Burns DK Roth J Shuldiner AR Molecular scanning of the human peroxisome proliferator activated receptor gamma (hPPAR gamma) gene in diabetic Caucasians: identification of a Pro12Ala PPAR gamma 2 missense mutation Biochem Biophys Res Commun 1997 241 270 274 10.1006/bbrc.1997.7798 9425261

[B16] Stumvoll M Häring H The peroxisome proliferator-activated receptor-gamma2 Pro12Ala polymorphism Diabetes 2002 51 2341 2347 10.2337/diabetes.51.8.2341 12145143

[B17] Mori H Ikegami H Kawaguchi Y Seino S Yokoi N Takeda J Inoue I Seino Y Yasuda K Hanafusa T The Pro12 Ala substitution in PPAR-gamma is associated with resistance to development of diabetes in the general population: possible involvement in impairment of insulin secretion in individuals with type 2 diabetes Diabetes 2001 50 891 894 10.2337/diabetes.50.4.891 11289058

[B18] Altshuler D Hirschhorn JN Klannemark M Lindgren CM Vohl MC Nemesh J Lane CR Schaffner SF Bolk S Brewer C Tuomi T Gaudet D Hudson TJ Daly M Groop L Lander ES The common PPARgamma Pro12Ala polymorphism is associated with decreased risk of type 2 diabetes Nat Genet 2000 26 76 80 10.1038/79216 10973253

[B19] Sanghera DK Ortega L Han S Singh J Ralhan SK Wander GS Mehra NK Mulvihill JJ Ferrell RE Nath SK Kamboh MI Impact of nine common type 2 diabetes risk polymorphisms in Asian Indian Sikhs: PPARG2 (Pro12Ala), IGF2BP2, TCF7L2 and FTO variants confer a significant risk BMC Med Genet 2008 9 59 18598350 10.1186/1471-2350-9-59 PMC2481250

[B20] Wang X Liu J Ouyang Y Fang M Gao H Liu L The association between the Pro12Ala variant in the PPARc2 gene and type 2 diabetes mellitus and obesity in a Chinese population PLoS ONE 2013 8 8 e71985 doi:10.1371/journal.pone.0071985 10.1371/journal.pone.0071985 23991018 PMC3749141

[B21] Burguete-Garcia AI Cruz-Lopez M Madrid-Marina V Lopez-Ridaura R Hernández-Avila M Cortina B Gómez RE Velasco-Mondragón E Association of Gly972Arg polymorphism of *IRS1* gene with type 2 diabetes mellitus in lean participants of a national health survey in Mexico: a candidate gene study Metabolism 2010 59 38 45 10.1016/j.metabol.2009.07.007 19716569

[B22] Erasmus RT Soita DJ Hassan MS Blanco Blanco E Vergotine Z Kengne AP Matsha TE High prevalence of diabetes mellitus and metabolic syndrome in a south african mixed ancestry population: The Bellville-South Africa study - baseline data S Afr Med J 2012 102 841 844 23116739 10.7196/samj.5670

[B23] Almind K Inoue G Pedersen O Kahn CR A common amino acid polymorphism in insulin receptor substrate-1 causes impaired insulin signaling. Evidence from transfection studies J Clin Invest 1996 97 2569 2575 10.1172/JCI118705 8647950 PMC507343

[B24] Prudente S Morini E Trischitta V Insulin signaling regulating genes: effect on T2DM and cardiovascular risk Nat Rev Endocrinol 2009 5 682 693 10.1038/nrendo.2009.215 19924153

[B25] Federici M Pandolfi A De Filippis EA Pellegrini G Menghini R Lauro D Cardellini M Romano M Sesti G Lauro R Consoli A G972R IRS-1 variant impairs insulin regulation of endothelial nitric oxide synthase in cultured human endothelial cells Circulation 2004 109 399 405 10.1161/01.CIR.0000109498.77895.6F 14707024

[B26] Voight BF Scott LJ Steinthorsdottir V Morris AP Dina C Welch RP Zeggini E Huth C Aulchenko YS Thorleifsson G McCulloch LJ Ferreira T Grallert H Amin N Wu G Willer CJ Raychaudhuri S McCarroll SA Langenberg C Hofmann OM Dupuis J Qi L Segrè AV van Hoek M Navarro P Ardlie K Balkau B Benediktsson R Bennett AJ Blagieva R Twelve type 2 diabetes susceptibility loci identified through large-scale association analysis Nat Genet 2010 42 579 589 10.1038/ng.609 20581827 PMC3080658

[B27] Rung J Cauchi S Albrechtsen A Shen L Rocheleau G Cavalcanti-Proença C Bacot F Balkau B Belisle A Borch-Johnsen K Charpentier G Dina C Durand E Elliott P Hadjadj S Järvelin MR Laitinen J Lauritzen T Marre M Mazur A Meyre D Montpetit A Pisinger C Posner B Poulsen P Pouta A Prentki M Ribel-Madsen R Ruokonen A Sandbaek A Genetic variant near IRS1 is associated with type 2 diabetes, insulin resistance and hyperinsulinemia Nat Genet 2009 41 1110 1115 10.1038/ng.443 19734900

[B28] Stumvoll M Stefan N Fritsche A Madaus A Tschritter O Koch M Machicao F Häring H Interaction effect between common polymorphisms in PPARgamma2 (Pro12Ala) and insulin receptor substrate 1 (Gly972Arg) on insulin sensitivity J Mol Med (Berl) 2002 80 33 38 10.1007/s001090100282 11862322

[B29] Mousavinasab F Tähtinen T Jokelainen J Koskela P Vanhala M Oikarinen J Keinänen-Kiukaanniemi S Laakso M Common polymorphisms in the PPARgamma2 and IRS1 genes and their interaction influence serum adiponectin concentration in young Finnish men Mol Genet Metab 2005 84 344 348 10.1016/j.ymgme.2004.11.008 15781195

[B30] Weyer C Funahashi T Tanaka S Hotta K Matsuzawa Y Pratley RE Tataranni PA Hypoadiponectinemia in obesity and type 2 diabetes: close association with insulin resistance and hyperinsulinemia J Clin Endocrinol Metab 2001 86 1930 1935 11344187 10.1210/jcem.86.5.7463

[B31] von Eynatten M Hamman A Twardella D Nawroth PP Brenner H Rothenbacher D Relationships of adiponectin with markers of systemic inflammation, Atherogenic dyslipidaemia, and heart failure in patients with coronary heart disease Clin Chem 2006 52 853 59 10.1373/clinchem.2005.060509 16556684

[B32] Hasstedt SJ Ren QF Teng K Elbein SC Effect of the peroxisome proliferator-activated receptor-gamma 2 pro(12)ala variant on obesity, glucose homeostasis, and blood pressure in members of familial type 2 diabetic kindreds J Clin Endocrinol Metab 2001 86 536 41 11158005 10.1210/jcem.86.2.7205

[B33] Stefan N Fritsche A Häring H Stumvoll M Effect of experimental elevation of free fatty acids on insulin secretion and insulin sensitivity in healthy carriers of the Pro12Ala polymorphism of the peroxisome proliferator–activated receptor-gamma2 gene Diabetes 2001 50 1143 1148 10.2337/diabetes.50.5.1143 11334419

[B34] de Wit E Delport W Rugamika CE Meintjes A Möller M van Helden PD Seoighe C Hoal EG Genome-wide analysis of the structure of the South African coloured population in the Western Cape Hum Genet 2010 14 145 153 20490549 10.1007/s00439-010-0836-1

[B35] Hancox RJ Landhuis CE Correlation between measures of insulin resistance in fasting and non-fasting blood Diabetol Metab Syndr 2011 3 23 10.1186/1758-5996-3-23 21899745 PMC3177770

[B36] Deng HW Population admixture may appear to mask, change or reverse genetic effects of genes underlying complex traits Genetics 2001 159 1319 1323 11729172 10.1093/genetics/159.3.1319 PMC1461858

[B37] Matsha TE Hassan MS Kidd M Erasmus RT The 30-year cardiovascular risk profile of South Africans with diagnosed diabetes, undiagnosed diabetes, pre-diabetes or normoglycaemia: the Bellville, South Africa pilot study Cardiovasc J Afr 2012 23 5 11 10.5830/CVJA-2010-087 22331244 PMC3721868

[B38] Chalmers J MacMahon S Mancia G Whitworth J Beilin L Hansson L Neal B Rodgers A Ni Mhurchu C,CT Clark T World health organization-international society of hypertension guidelines for the management of hypertension. Guidelines sub-committee of the world health organization Clin Exp Hypertens 1999 21 1009 1060 10423121 10.3109/10641969909061028

[B39] Alberti KG Zimmet PZ Definition, diagnosis and classification of diabetes mellitus and its complications. Part 1: diagnosis and classification of diabetes mellitus provisional report of a WHO consultation Diabet Med 1998 15 539 553 10.1002/(SICI)1096-9136(199807)15:7<539::AID-DIA668>3.0.CO;2-S 9686693

[B40] Friedewald WT Levy RI Fredrickson DS Estimation of the concentration of low-density lipoprotein cholesterol in plasma, without use of the preparative ultracentrifuge Clin Chem 1972 18 499 502 4337382

[B41] Levey AS Bosch JP Lewis JB Greene T Rogers N Roth D Modification of Diet in Renal Disease Study Group A more accurate method to estimate glomerular filtration rate from serum creatinine: a new prediction equation Ann Intern Med 1999 130 461 470 10.7326/0003-4819-130-6-199903160-00002 10075613

[B42] Levey AS Coresh J Greene T Stevens LA Zhang YL Hendriksen S Kusek JW Van Lente F Chronic Kidney Disease Epidemiology Collaboration Using standardized serum creatinine values in the modification of diet in renal disease study equation for estimating glomerular filtration rate Ann Intern Med 2006 145 247 254 10.7326/0003-4819-145-4-200608150-00004 16908915

